# Spectrum of Genitourinary Dysgenesis on Magnetic Resonance Imaging: A Case Series of Zinner and Mayer-Rokitansky-Küster-Hauser Syndromes

**DOI:** 10.7759/cureus.107993

**Published:** 2026-04-29

**Authors:** Varun S Trivedi, Narendra G Tembhekar, Varsha Rathi, Nitin R Bhutada, Amit G Disawal, Ramesh Parate

**Affiliations:** 1 Department of Radiology, Government Medical College and Hospital, Nagpur, IND

**Keywords:** genitourinary anomalies, magnetic resonance imaging, mrkh syndrome, pelvic mri, primary amenorrhea, renal agenesis, seminal vesicle cyst, zinner syndrome

## Abstract

Congenital anomalies of the genitourinary system are uncommon but clinically significant due to their impact on urinary and reproductive function. Zinner syndrome and Mayer-Rokitansky-Küster-Hauser (MRKH) syndrome are rare developmental disorders involving the mesonephric and Müllerian ducts, respectively, and often present with nonspecific clinical features, making imaging essential for accurate diagnosis.

We present a series of three cases evaluated using magnetic resonance imaging (MRI). One case involved a young male presenting with dysuria and testicular discomfort, demonstrating unilateral renal agenesis, an ipsilateral seminal vesicle cyst, and an associated atretic ureter, consistent with Zinner syndrome. The other two cases involved young females presenting with primary amenorrhea. MRI revealed the absence of the uterus and upper vagina with normal ovaries in both cases, consistent with MRKH syndrome, with one case showing associated bilateral ectopic kidneys.

MRI provided a comprehensive evaluation of pelvic anatomy, clearly delineating the congenital anomalies and associated findings. Recognition of characteristic imaging features is essential for accurate diagnosis and appropriate clinical management.

## Introduction

Congenital anomalies of the genitourinary system arise from disturbances in the development of the mesonephric (Wolffian) and paramesonephric (Müllerian) ducts. Although relatively uncommon, these anomalies are clinically significant due to their impact on urinary and reproductive function and often present with nonspecific symptoms, making imaging essential for accurate diagnosis [1,2].

Mayer-Rokitansky-Küster-Hauser (MRKH) syndrome results from failure of Müllerian duct development and is a well-recognized cause of primary amenorrhea in females with normal secondary sexual characteristics [3,4]. It is broadly classified into type A, involving isolated uterovaginal agenesis, and type B, which is associated with additional anomalies, most commonly involving the renal system [5].

Zinner syndrome is a rare congenital condition characterized by the triad of unilateral renal agenesis, an ipsilateral seminal vesicle cyst, and ejaculatory duct obstruction, resulting from abnormal development of the mesonephric duct [6,7]. Patients typically present in early adulthood with symptoms such as dysuria, perineal discomfort, or infertility.

Magnetic resonance imaging (MRI) plays a pivotal role in the evaluation of these anomalies due to its excellent soft tissue contrast and multiplanar capability. It allows precise delineation of pelvic anatomy, assessment of associated abnormalities, and accurate characterization of congenital malformations [1,3].

In this case series, we present the MRI spectrum of Zinner syndrome and MRKH syndrome, highlighting their characteristic imaging features and associated findings.

## Case presentation

Case 1

**Figure 1:**
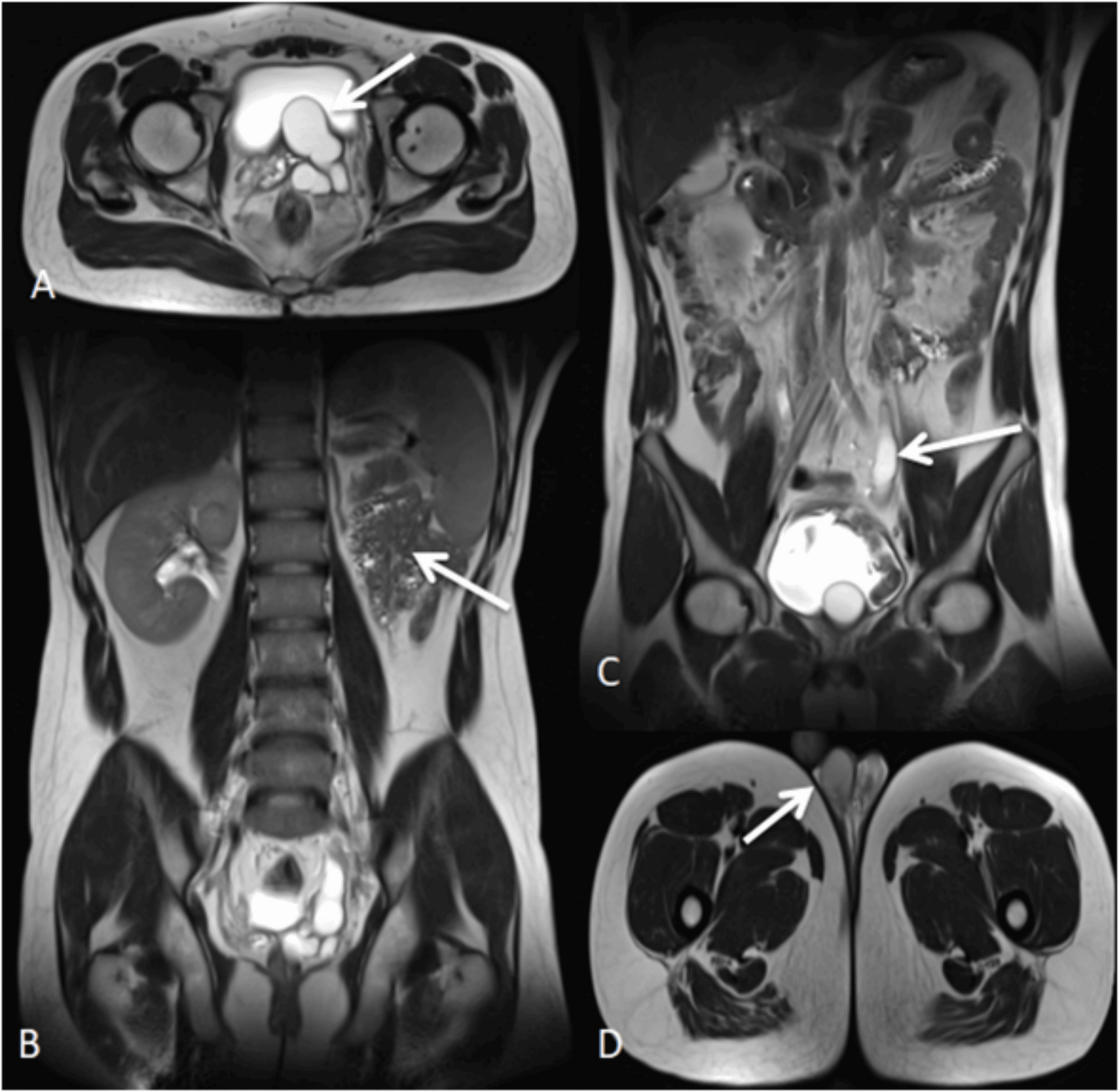
Zinner syndrome imaging spectrum (A) Axial T2-weighted HASTE image showing an enlarged left seminal vesicle with multiple hyperintense cystic areas (white arrow), suggestive of seminal vesicle cysts. (B) Coronal T2-weighted HASTE image demonstrating non-visualization of the left kidney (white arrow), consistent with left renal agenesis. (C) Coronal T2-weighted HASTE image demonstrating a dilated, tortuous cystic tubular structure in the pelvis (white arrow) communicating with the left seminal vesicle. (D) Axial T2-weighted HASTE image showing the right testis within the scrotum (white arrow). HASTE: Half-Fourier-acquired single-shot turbo spin echo.

A 26-year-old male presented with complaints of pain during micturition and left testicular discomfort for two months. MRI revealed non-visualization of the left kidney, consistent with renal agenesis, and an enlarged left seminal vesicle with multiple cystic areas. A dilated, tortuous, blind-ending cystic tubular structure was noted in the pelvis communicating with the seminal vesicle, suggestive of an atretic ureter. These findings were consistent with Zinner syndrome (Figure 1). This constellation of findings can be explained by abnormal development of the mesonephric (Wolffian) duct, which gives rise to the ureteric bud, seminal vesicle, and ejaculatory duct, thereby resulting in the characteristic triad of Zinner syndrome [6].

Case 2

**Figure 2:**
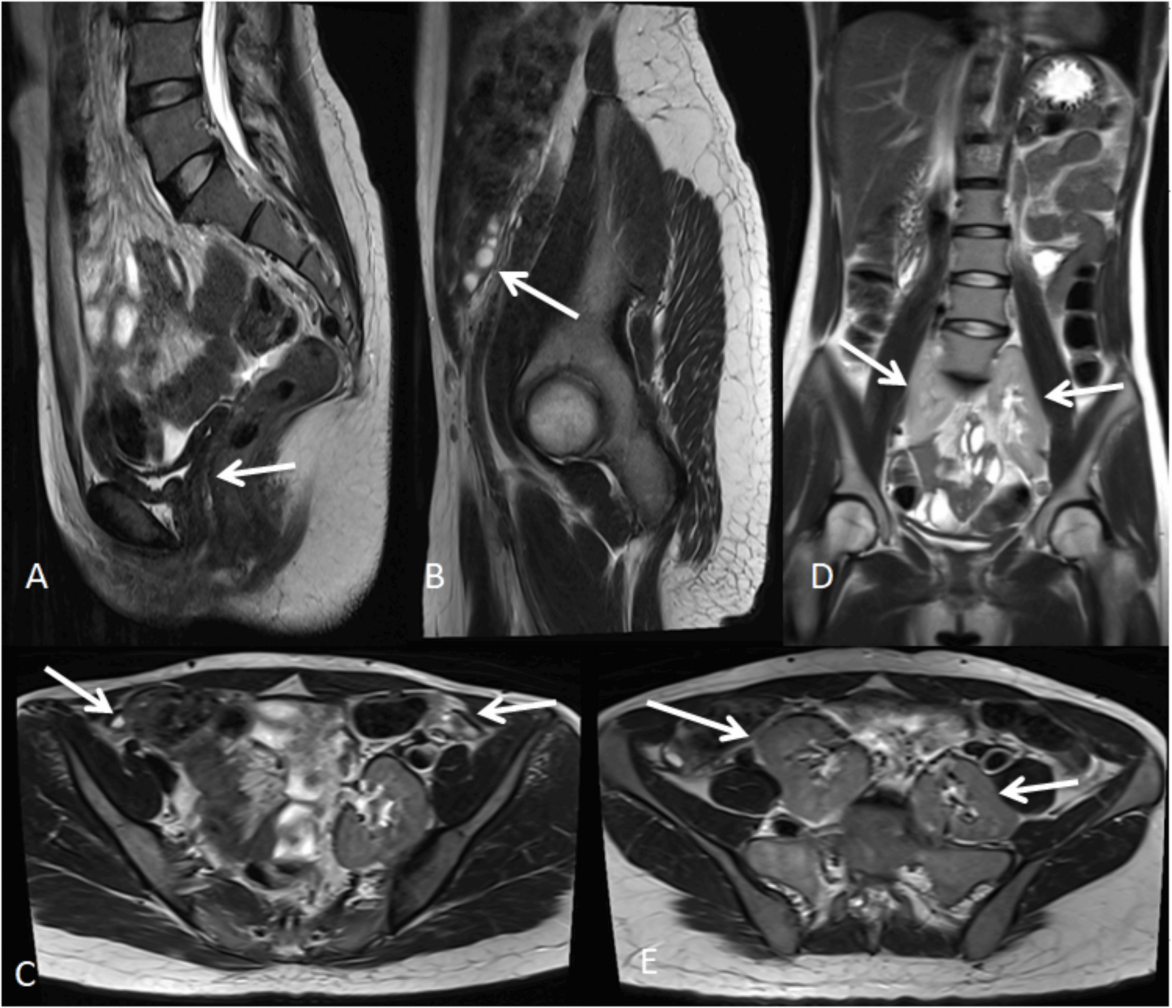
Mayer-Rokitansky-Küster-Hauser syndrome type B (A) Sagittal T2-weighted image demonstrating the absence of the uterus, cervix, and upper two-thirds of the vagina (white arrow), consistent with Müllerian agenesis. (B) Sagittal T2-weighted image showing normal-appearing ovaries with follicles (white arrow). (C) Axial T2-weighted image confirming normal bilateral ovaries (white arrows). (D) Coronal T2-weighted HASTE image showing the absence of kidneys in the renal fossae (white arrows) with ectopic pelvic kidneys. (E) Axial T2-weighted image demonstrating bilateral ectopic kidneys in the presacral region with inferomedially directed hila (white arrows). HASTE: Half-Fourier-acquired single-shot turbo spin echo.

A 22-year-old female presented with primary amenorrhea. MRI demonstrated the absence of the uterus, cervix, and upper two-thirds of the vagina, consistent with Müllerian agenesis. Bilateral kidneys were not visualized in the renal fossae and were instead located in the presacral pelvic region, indicating bilateral ectopic kidneys. Both ovaries appeared normal with developing follicles. These findings were consistent with MRKH syndrome (American Society for Reproductive Medicine (ASRM) Class I) with associated renal anomalies (Figure 2) [3,5]. These findings reflect failure of development of the paramesonephric (Müllerian) ducts, which form the uterus, cervix, and upper vagina, while sparing the ovaries that arise from the gonadal ridge [3].

Case 3

**Figure 3:**
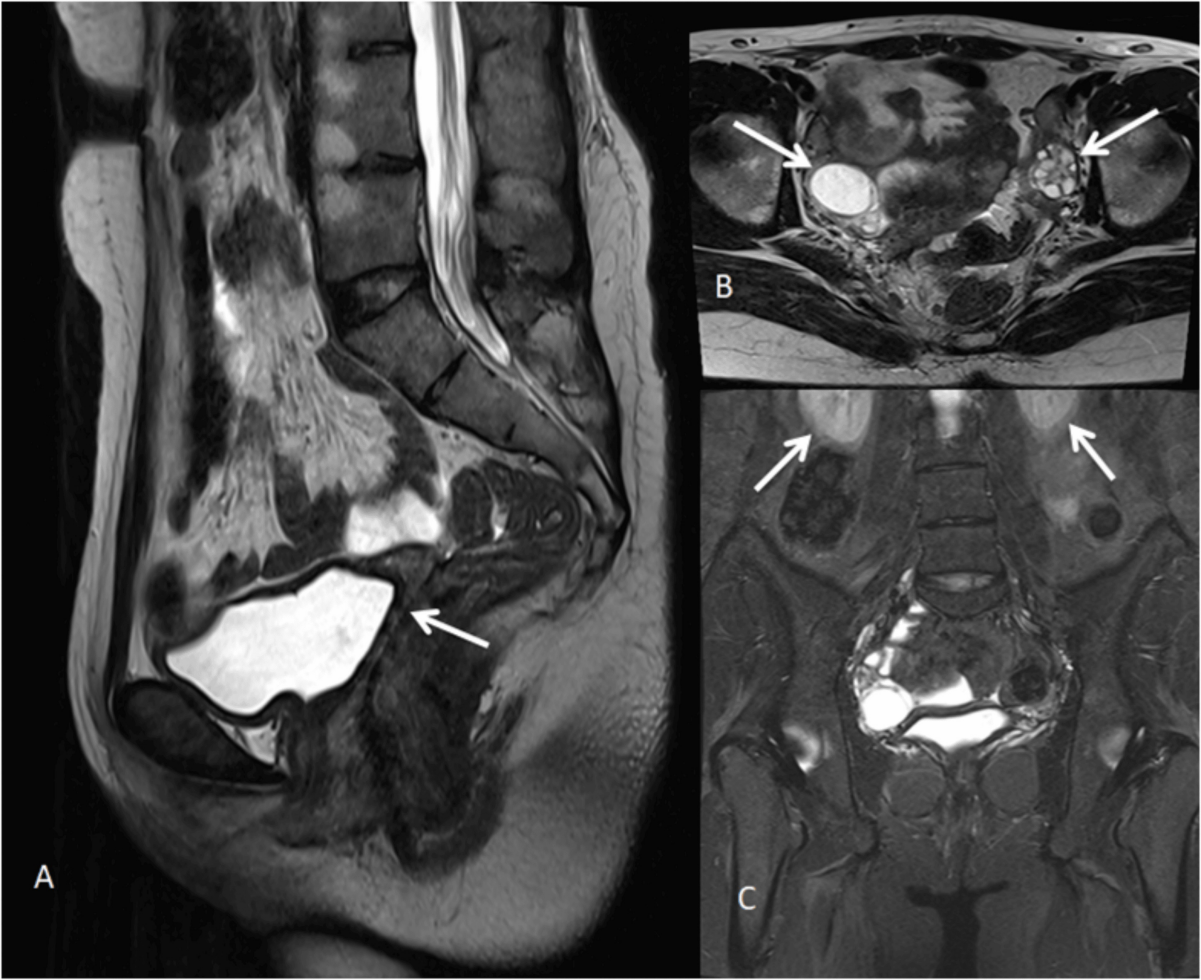
Mayer-Rokitansky-Küster-Hauser syndrome type A (A) Sagittal T2-weighted image demonstrating the absence of the uterus, cervix, and upper two-thirds of the vagina (white arrow), consistent with Müllerian agenesis. (B) Axial T2-weighted image showing normal bilateral ovaries with follicles (white arrows). (C) Coronal STIR image demonstrating normally located kidneys in the renal fossae (white arrows). STIR: Short tau inversion recovery.

A 22-year-old female presented with primary amenorrhea and a history of atrial septal defect closure. MRI revealed the absence of the uterus, cervix, and upper two-thirds of the vagina. A thin linear hypointense band extending toward the perineum was noted, suggestive of an atretic vagina. The ovaries appeared normal with developing follicles. These findings were consistent with MRKH syndrome (ASRM Class I) (Figure 3). These findings reflect failure of development of the paramesonephric (Müllerian) ducts, which form the uterus, cervix, and upper vagina, while sparing the ovaries that arise from the gonadal ridge [3].

## Discussion

Congenital anomalies of the genitourinary system arise from disturbances in the development of the mesonephric (Wolffian) and paramesonephric (Müllerian) ducts and encompass a wide spectrum of structural abnormalities [1,2]. An understanding of this developmental basis is important for accurate interpretation of imaging findings and appropriate clinical management.

Zinner syndrome is a rare congenital anomaly resulting from abnormal development of the mesonephric duct, classically presenting with the triad of unilateral renal agenesis, an ipsilateral seminal vesicle cyst, and ejaculatory duct obstruction [6,7]. Patients are often asymptomatic in early life and typically present in the second or third decade with nonspecific symptoms such as dysuria, perineal pain, or infertility. Imaging plays a crucial role in diagnosis, with MRI providing excellent soft tissue characterization and anatomical delineation. The characteristic findings include cystic dilatation of the seminal vesicle, absence of the ipsilateral kidney, and a dilated or atretic ureter, as demonstrated in our case.

MRKH syndrome results from the failure of Müllerian duct development and is a well-recognized cause of primary amenorrhea in females with normal secondary sexual characteristics [3,5]. It is broadly classified into type A, which involves isolated uterovaginal agenesis, and type B, which is associated with additional anomalies, most commonly involving the renal system [4]. MRI is the modality of choice for evaluation, allowing detailed assessment of uterine absence, vaginal anatomy, and ovarian morphology, as well as detection of associated renal anomalies, as illustrated in our cases [1,3].

Differentiation between these entities is important due to differences in clinical presentation, management, and prognosis. While Zinner syndrome primarily affects males and is related to mesonephric duct abnormalities, MRKH syndrome affects females and involves Müllerian duct agenesis. MRI provides a comprehensive, non-invasive evaluation of pelvic anatomy and associated abnormalities, making it indispensable in the assessment of these congenital conditions.

## Conclusions

MRI plays a crucial role in the evaluation of congenital genitourinary anomalies by providing detailed anatomical delineation and identifying associated abnormalities. Recognition of characteristic imaging features of conditions such as Zinner syndrome and MRKH syndrome enables accurate diagnosis and appropriate clinical management. Early and precise imaging assessment is essential for guiding treatment decisions and improving patient outcomes.
